# Recent Advances in Phase-Change-Coupled Interfacial Evaporation: Thermal-Mass Management and Multifunctional Applications

**DOI:** 10.3390/nano16161010

**Published:** 2026-08-17

**Authors:** Xinshuo Li, Qian Chen, Xiaoke Li

**Affiliations:** 1 College of Intelligent Manufacturing and Automobile, Chengdu Vocational & Technical College of Industry, Chengdu 610213, China; 2 College of Materials and Chemistry & Chemical Engineering, Chengdu University of Technology, Chengdu 610059, China; chenqian001008@126.com

**Keywords:** solar-driven interfacial evaporation, phase change materials, thermal-mass management, all-weather desalination, multifunctional system integration

## Abstract

Solar-driven interfacial evaporation (SDIE) represents a highly promising technology for decentralized desalination and wastewater treatment, yet its practical industrial deployment is severely constrained by the intrinsic intermittency of natural solar irradiance and nocturnal salt crystallization. To smooth energy fluctuations and achieve all-weather, continuous freshwater output, integrating solid–liquid phase change materials (SLPCMs) into SDIE has evolved into a system-level paradigm shift driven by advanced spatiotemporal thermal-mass management. This review systematically summarizes recent breakthroughs in micro-to-macro structural engineering for phase-change-coupled SDIE systems. Spatially, advanced microscopic encapsulation strategies such as 3D matrices, core–shell architectures, and solid–solid transitions eradicate molten PCM leakage and reconstruct heat transfer networks, while macroscopic configurations involving sandwich structures and 3D directional channels realize functional zoning to maximize thermal localization. Temporally, the controlled nocturnal release of stored latent heat establishes a cross-timeline energy relay, sustaining dark evaporation and activating interfacial hydrodynamics via Marangoni convection and thermophoretic diffusion to prevent salt clogging under extreme conditions. Furthermore, cross-disciplinary integrations for water-electricity co-generation, targeted resource recovery, and environmental remediation are comprehensively discussed. Finally, critical engineering challenges regarding scalability, cost-effectiveness, and condensation system integration are addressed, offering forward-looking perspectives on coupling thermal storage with physical catalysis to transcend classical thermodynamic limits.

## 1. Introduction

The escalating scarcity of freshwater resources has emerged as a critical global challenge severely constraining the sustainable development of human society [1,2,3,4,5,6]. Among various water purification methodologies, solar-driven interfacial evaporation (SDIE) has attracted significant attention as a highly promising decentralized strategy for seawater desalination and wastewater treatment owing to its low carbon footprint, zero consumption of conventional fossil fuels, minimal capital investment, and exceptional eco-friendliness [7,8,9,10,11]. Distinct from conventional bulk-heating systems, the cornerstone of SDIE lies in its “thermal localization” mechanism. By floating a meticulously engineered photothermal material at the water–air interface, the system can precisely convert absorbed broadband solar radiation into localized thermal energy, strictly confining it within a micron-thick surface water film to achieve an outstanding light-to-steam conversion efficiency [12,13,14,15]. However, the practical industrial deployment of SDIE technology is still hindered by a fatal physical bottleneck: the intrinsic intermittency and uncontrollable fluctuations of natural solar irradiance [16,17]. Under real-world outdoor operating conditions, solar radiation intensity is inevitably subject to severe interference from natural meteorological dynamics, such as diurnal cycles, cloud shading, and overcast or rainy weather. Lacking an effective energy buffering mechanism, conventional SDIE systems exhibit a freshwater production rate that is highly coupled and synchronously oscillating with real-time solar intensity [18,19,20]. Once the sun sets or solar intensity suddenly fades, the interfacial thermal localization effect vanishes immediately, causing the evaporator surface temperature to rapidly drop to ambient levels and leading to a complete cessation of the water production process [21,22]. This intermittent operational mode not only severely restricts the daily cumulative freshwater yield but also easily triggers excessive salt concentration and crystallization on the evaporator surface during nocturnal hours, which clogs the porous water transport channels and severely undermines the long-term stability and operational continuity of the device [23,24,25,26]. Consequently, suppressing the spatiotemporal fluctuations of solar energy to achieve all-weather, highly efficient, and continuous freshwater output has become a critical and universal scientific challenge in the field of solar-thermal interfacial evaporation.

To overcome the energy supply bottleneck caused by solar intermittency, researchers have attempted to integrate thermal energy storage technologies into SDIE systems [27,28]. Among various thermal storage media, solid–liquid phase change materials (SLPCMs) have become ideal candidates for smoothing energy fluctuations due to their outstanding latent heat storage capacity at specific temperatures, high chemical stability, and tailorable phase transition temperature windows [29]. Reviewing the technological evolution, the integration of PCMs with interfacial evaporators has progressed from disordered physical stacking to sophisticated micro-to-macro structural engineering. In the early exploration stage, researchers generally adopted simple physical blending or plug-in configurations, where bulk PCMs were placed directly beneath the photothermal material or the water-wicking substrate [30,31]. The fundamental working mechanism relied on downward heat conduction, where excess waste heat generated by the top photothermal layer melted the PCM to store heat during periods of strong daytime illumination; in the absence of light at night, the PCM solidified and released latent heat to backwardly feed the interfacial water for sustained evaporation [32]. However, such primitive physical configurations rapidly exposed severe design deficiencies under practical operating conditions: first, macroscopic liquid leakage and environmental contamination occurred as the solid–liquid PCMs experienced a dramatic surge in fluidity upon melting, easily leading to macroscopic leakage in open water treatment environments, which not only caused rapid degradation of thermal storage capacity but also resulted in secondary water pollution [33,34,35]. Second, the thermal resistance network was severely deteriorated because conventional organic PCMs possess extremely low intrinsic thermal conductivity; placing a dense bulk PCM underneath prolonged the thermal response time of the system and hindered the temperature rise of the evaporation surface during the day. Finally, mass transport was severely impeded, as unstructured PCMs blocked the vertical capillary wicking pathways for water molecules and the escape routes for generated vapor, plunging the system into a dilemma of sufficient heat but dried-up water supply. These early bottlenecks demonstrate that simple physical stacking of materials fails to achieve efficient energetic synergy.

**Figure 1:**
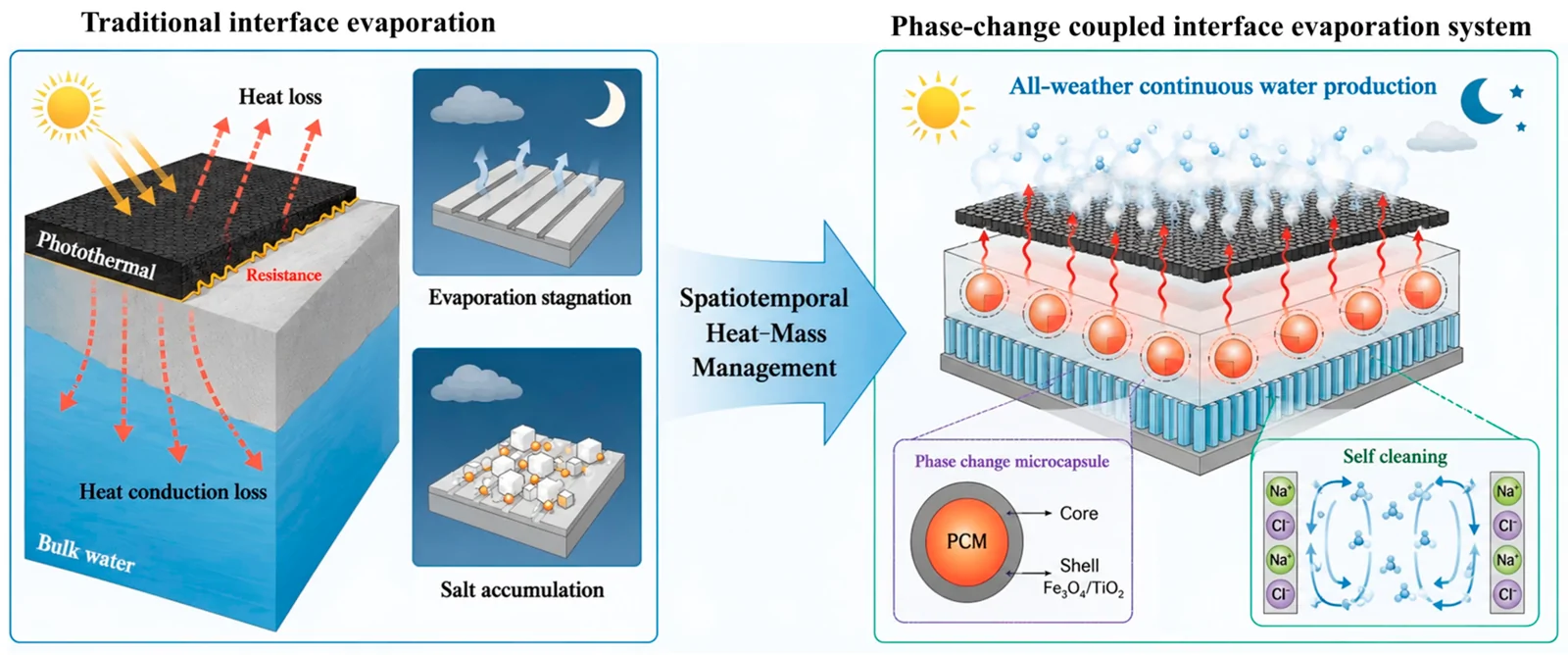
Schematic illustration comparing traditional interfacial evaporation and the phase-change-coupled system for all-weather continuous freshwater production and self-cleaning anti-salt fouling via spatiotemporal thermal-mass management.

Based on the aforementioned developmental bottlenecks, this review proposes that the introduction of PCMs into solar-driven interfacial evaporation is by no means a simple thermal storage add-on, but rather a profound paradigm shift at the system engineering level. Recent cutting-edge studies indicate that through multiscale microscopic material encapsulation and macroscopic structural engineering, PCM-coupled systems can fundamentally decouple heat and mass transfer in both spatial and temporal dimensions from a thermodynamic perspective. This sophisticated spatiotemporal thermal-mass management reconfigures the energy and material flow throughout the entire system. In the spatial dimension, firmly anchoring the PCM via microscopic core–shell structures, 3D gel networks, or covalently bonded solid–solid phase transitions not only thoroughly eliminates leakage but also dramatically lowers the interfacial thermal resistance by integrating thermally conductive media; concurrently, macroscopic designs such as sandwich-structured configurations or 3D directional porous channels achieve functional zoning for water supply, photothermal conversion, and thermal storage, precisely localizing the latent heat at the evaporation interface. In the temporal dimension, the steady release of latent heat by the PCM during sunset or cloudy periods establishes a cross-timeline “energy relay”. This long-lasting microscopic temperature gradient further activates Marangoni convection and thermophoretic diffusion within the fluid boundary layer, fundamentally conquering the persistent issue of salt crystallization and clogging during extreme hypersaline wastewater treatment (Figure 1). More importantly, this outstanding and stable thermal-mass management platform serves as an underlying thermodynamic regulator, driving the system to transcend traditional boundaries toward comprehensive multifunctional applications, including simultaneous water-electricity co-generation and in-situ recovery of target resources. Grounded in these insights, this review strictly centers around the core theme of thermal-mass management, systematically summarizing the recent breakthroughs in microscopic encapsulation strategies and macroscopic structural engineering. Furthermore, it provides an in-depth analysis of the all-weather operational and anti-salt mass transfer mechanisms under extreme conditions, while outlining the frontiers of cross-disciplinary integration for multifunctional systems.

## 2. Microscopic Encapsulation and Thermal Resistance Reconfiguration

SLPCMs have emerged as an ideal candidate for smoothing the intermittent fluctuations of solar energy owing to their exceptional latent heat storage capacity. However, within open solar-driven interfacial evaporation systems, these materials are highly susceptible to macroscopic leakage when in their molten state. This not only results in a continuous degradation of the system’s thermal storage capacity but also poses a severe risk of secondary water contamination [36]. Furthermore, conventional SLPCMs suffer from an extremely low intrinsic thermal conductivity (typically <0.3 W·m^−1^·K^−1^) and large supercooling degrees, which significantly prolongs the thermal response time and establishes a substantial thermal resistance barrier [37]. Therefore, the development of advanced microscopic encapsulation technologies is not only an engineering prerequisite to maintain the long-term phase stability of the material but also a critical pivot to reconstruct the interfacial heat transfer network and enhance the global energy utilization efficiency from a microscopic perspective. Currently, prevailing microscopic modification strategies primarily focus on physical confinement within 3D matrices, core–shell microcapsule engineering, and chemically bonded solid–solid phase transitions [38].

**Figure 2:**
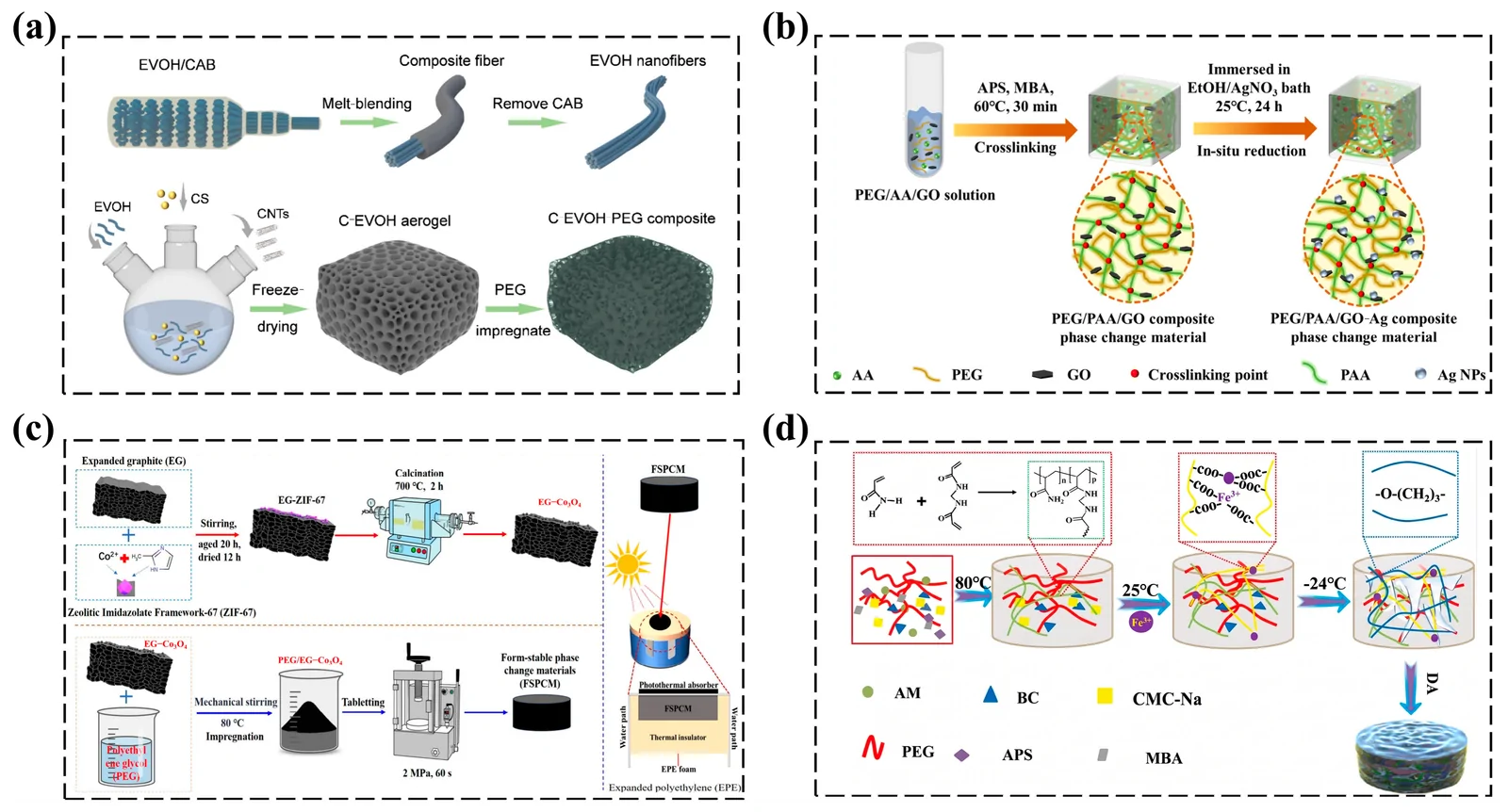
Microscopic encapsulation strategies of SLPCMs via 3D matrix physical confinement. (**a**) Fabrication process of shape-stabilized C-EVOH-PEG composite phase change materials based on EVOH nanofiber aerogels. Reprinted with permission from ref. [41]. Copyright 2025, Elsevier; (**b**) in-situ hybridization protocol of PEG/PAA/GO-Ag hydrogel composite networks with photothermal conductive fillers. Reprinted with permission from ref. [42]. Copyright 2025, Elsevier; (**c**) synthesis pathway of form-stable EG-Co_3_O_4_/PEG composites and the corresponding structural integration within a solar interfacial evaporator. Reprinted with permission from ref. [43]. Copyright 2025, Elsevier; (**d**) construction mechanism of AM/PEG/BC/CMC@PDA 3D blended gel networks for synergistic anti-leakage and thermal management. Reprinted with permission from ref. [44]. Copyright 2024, Wiley-VCH.

Physically confining SLPCMs within 3D porous architectures with high porosity and large specific surface areas, such as aerogels, hydrogels, and biomass networks, represents the most direct and efficient anti-leakage approach [39]. In this strategy, the intricate capillary forces within the porous matrix, coupled with the hydrogen bonding interactions between the matrix network and the phase change molecular chains, synergistically construct a dual material barrier to lock the liquid PCM. This methodology offers relatively simple fabrication processes, high cost-effectiveness, and excellent compatibility with diverse material compositions, geometries, and dimensions, while the incorporation of carbonaceous or metallic substrates can simultaneously compensate for the low thermal conductivity of the phase change media. Recent advancements indicate that polymer-based porous aerogels demonstrate exceptional shape stability while maintaining high thermal energy storage capacities [40]. For instance, Zhang et al. [41] developed a shape-stabilized composite phase change material (C-EVOH-PEG) based on poly (vinyl alcohol-co-ethylene) (EVOH) nanofiber aerogels and polyethylene glycol (PEG). Benefiting from the abundant porous channels of the C-EVOH aerogel and the robust hydrogen bonding and capillary synergy between the PEG molecular segments and EVOH nanofibers, the composite achieved an ultra-low leakage loss rate of only 1.77% after 20 melting-solidification cycles, while retaining a high energy storage density of up to 189.7 J·g^−1^ (Figure 2a). Nevertheless, pure physical confinement often introduces extra interfacial contact thermal resistance and fails to resolve the intrinsic lack of thermal conductivity within the PCM. To overcome this, synergistic strategies involving “one-pot” crosslinking or in-situ hybridization with highly conductive and light-absorbing fillers have emerged. Dong et al. [42] integrated graphene, silver nanoparticles (Ag NPs), or polydopamine (PDA) within a 3D network, which not only reconstructed internal heat transfer pathways but also endowed the matrix with prominent photothermal conversion features (Figure 2b). In their work, graphene oxide and silver nanoparticles were reduced in situ within a polyethylene glycol/poly(acrylic acid) (PEG/PAA/GO-Ag) hydrogel network, wherein the hydrogel served as a leak-proof support while the GO and Ag NPs acted as efficient photothermal conductive media, allowing the system to achieve a broadband solar absorption of 95.4%. This microscopic multi-component architecture accelerates latent heat capture and release due to the establishment of the conductive network, without sacrificing thermal storage capacity. Furthermore, Li et al. [43] confined PEG within an expanded graphite/cobalt tetroxide (EG-Co_3_O_4_) porous composite matrix. By leveraging the synergistic effects of skeleton adsorption and nanopore confinement, they fundamentally eradicated phase-change leakage. Owing to a significantly enhanced thermal conductivity (6.432 W·m^−1^·K^−1^) and a dramatically suppressed supercooling degree (from 15.7 °C to 9.6 °C), the light-to-steam conversion efficiency of the solar interfacial evaporator integrated with this PCM was successfully elevated from 58.6% to 80.3% (Figure 2c). Similarly, Dong et al. [44] constructed a 3D blended gel network using acrylamide (AM), bacterial nanocellsulose (BC), and sodium carboxymethyl cellulose (CMC-Na) (AM/PEG/BC/CMC@PDA), likewise achieving an efficient synergy between microscopic leakage prevention and global thermal management (Figure 2d).

**Figure 3:**
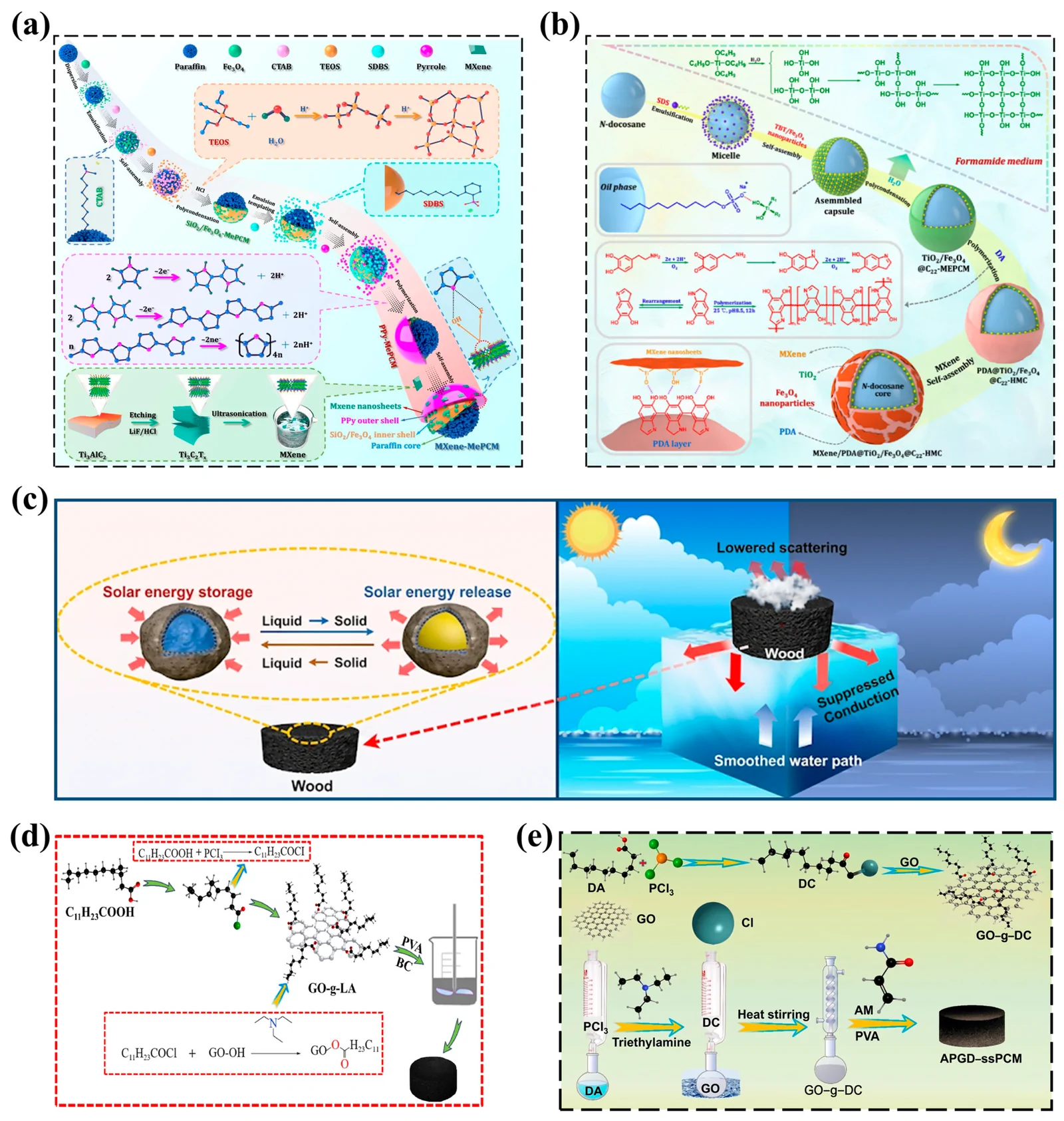
Advanced microscopic stabilization strategies of PCMs via core–shell microencapsulation and covalent solid–solid phase transitions. (**a**) Fabrication process of microcapsules with a SiO_2_/Fe_3_O_4_ composite shell encapsulating paraffin twin-cores, sequentially functionalized with PPy and MXene nanosheets. Reprinted with permission from ref. [47]. Copyright 2023, Elsevier; (**b**) stepwise synthesis protocol of MXene/PDA@TiO_2_/Fe_3_O_4_@C_22_-HMC microcapsules featuring magnetic/photothermal dual-functional shells and an n-docosane core. Reprinted with permission from ref. [48]. Copyright 2022, American Chemical Society; (**c**) working mechanism of PDA@JPs@PCM demonstrating solar energy storage/release loops. Reprinted with permission from ref. [49]. Copyright 2026, Elsevier; (**d**) molecular configuration and grafting mechanism of leak-proof solid–solid BC/PVA/GO-g-LA-SSPCMs. Reprinted with permission from ref. [50]. Copyright 2024, Elsevier; (**e**) chemical synthesis route of APGD-ssPCM solid–solid phase change hydrogels combining efficient photothermal storage with rapid hydrophilic water transport pathways. Reprinted with permission from ref. [51]. Copyright 2025, Elsevier.

Beyond macroscopic matrix confinement, core–shell microencapsulation technology isolates PCMs within a closed “shell” composed of inorganic, organic, or hybrid materials, thereby completely segregating the PCMs from the external fluid environment in physical space [45]. Within a phase-change-coupled evaporation system, the structural design of the microcapsule shell not only assumes the responsibility of structural leakage prevention but is also endowed with multifunctional properties such as broadband absorption, reduced supercooling, and controllable recyclability. To overcome the low energy utilization efficiency of traditional single-component microcapsule evaporators under unstable illumination, the reconstruction of multifunctional shells has become a research hotspot [46]. Magnetic nanoparticles are frequently introduced into inorganic shells to improve the thermodynamic stability of the microcapsules. Liu et al. [47] fabricated microcapsules featuring a SiO_2_/Fe_3_O_4_ composite shell encapsulating twin phase-change cores of n-tetracosane and n-eicosane, followed by surface modification with polypyrrole (PPy) and MXene nanosheets. Within this hierarchical architecture, the hollow inorganic shell effectively blocked liquid PCM leakage, while the integrated Fe_3_O_4_ nanoparticles provided abundant nucleation sites for PCM crystallization, thereby significantly weakening the supercooling phenomenon during phase transition (Figure 3a). More importantly, the introduction of the magnetic shell endows the material with robust magnetic separation capabilities, enabling the evaporator to be easily separated from accumulated salt crystals under an external magnetic field, which greatly enhances the long-term cyclic lifetime of the system. In a similar study, Zheng et al. [48] employed a TiO_2_/Fe_3_O_4_ shell to encapsulate n-docosane, with subsequent epitaxial modification of PDA/MXene on its surface, achieving a high spectral absorption of over 95% and a remarkable enhancement in thermal conductivity (Figure 3b). Concurrently, the introduction of the Fe_3_O_4_ shell imparted excellent magnetic separation and cyclic washing reuse stability, while the latent heat release from the phase-change core boosted the total water yield by 1.17 kg m^−2^ on cloudy days, effectively overcoming the intermittent drawbacks of solar energy. Additionally, manipulating the microscopic orientation and wettability of the microcapsules can further optimize the mass transfer resistance at the phase-change interface. Ma et al. [49] developed an evaporator featuring Janus particle-modified natural wood (PDA@JPs@PCM), where octadecane was precisely encapsulated within the silica-based Janus microcapsules by low-surface-energy polymer ends (PS/PDVB), while the other end bearing silicon hydroxyl groups was tightly bonded to PDA via hydrogen bonds (Figure 3c). This unique asymmetric wettability microcapsule structure ensures long-term leak-proof performance of the phase-change layer on the one hand and dramatically minimizes the mass transfer resistance of water molecules transported to the evaporation interface on the other hand, providing microscopic kinetic support for all-weather, highly efficient evaporation.

Although 3D matrix confinement and core–shell encapsulation have made significant progress in preventing PCM leakage, physical barriers still confront limitations under long-term high-low temperature thermal cycling and extreme water quality conditions; once the shell ruptures or the porous channels disintegrate, leakage remains inevitable. To thoroughly eradicate the generation of the liquid phase, utilizing chemical modification to transform conventional solid–liquid PCMs into solid–solid PCMs has emerged as the ultimate solution at the material level. The core mechanism of solid–solid phase transitions relies on covalent bonding to chemically graft phase-change functional segments as side chains or blocks onto a rigid skeleton material. Upon heating, the phase-change segments only undergo transitions between crystalline and amorphous states, while the entire material consistently maintains a robust solid state macroscopically. For instance, Dong et al. [50] successfully fabricated BC/PVA/GO-g-LA-SSPCMs composite materials by chemically grafting lauric acid (LA) and graphene oxide (GO), combined with modifying a bacterial cellulose (BC) substrate with polyvinyl alcohol (PVA) (Figure 3d). Within this molecular-level composite network, LA acts as the phase-change unit firmly anchored by rigid chemical bonds, demonstrating ultra-high structural stability with zero leakage and zero secondary contamination even after experiencing 15 extreme light–dark alternating thermal cycles and being exposed to harsh environments such as strong acids, strong alkalis, and dye wastewater. Moreover, the chemical grafting method can isomorphicize the thermal storage function with specific hydrophilic transport pathways. Dong et al. [51] synthesized a solid–solid phase change thermal energy storage gel (APGD-ssPCM) via a grafting approach, which achieved a high broadband solar absorption rate of 96.2% while establishing an outstanding equilibrium swelling water content of 104.83 g·g^−1^ and a rapid water transport rate of 41.93 g·g^−1^·min^−1^ through the abundant active sites introduced by grafting (Figure 3e). This design, starting from the molecular configuration, fundamentally breaks the intrinsic contradiction between thermal storage without water conduction and water conduction with easy leakage typical of conventional PCMs. By eliminating liquid-phase fluidity, it not only significantly reduces the microscopic thermal resistance but also guarantees the dual stability of both the structure and energy output of the entire evaporation system across spatiotemporal domains (Table 1).

## 3. Macroscopic Structural Design and Heat Flow Decoupling

Following the successful microscopic encapsulation of phase change materials and the concomitant reduction of their intrinsic thermal resistance, the rational layout of photothermal components, water transport pathways, and thermal storage units within the global device configuration stands as another pivotal determinant of system energy utilization efficiency. Conventional solar-driven interfacial evaporators typically employ a simplistic bilayer configuration [52]. Direct integration of a PCM into such structures frequently results in a sluggish surface temperature response or substantial thermal dissipation into the underlying bulk water, owing to the large thermal capacity and non-uniform thermal conductivity of the PCM. To circumvent this critical thermal loss bottleneck, researchers have pioneered macroscopic structural engineering strategies, developing sandwich-structured hierarchical configurations and 3D directional porous networks [53]. These innovative macroscopic designs achieve an organic decoupling of the “water supply, photothermal conversion, and thermal storage” functions in physical space, thereby precisely localizing the global heat flux at the gas–liquid evaporation interface.

**Figure 4:**
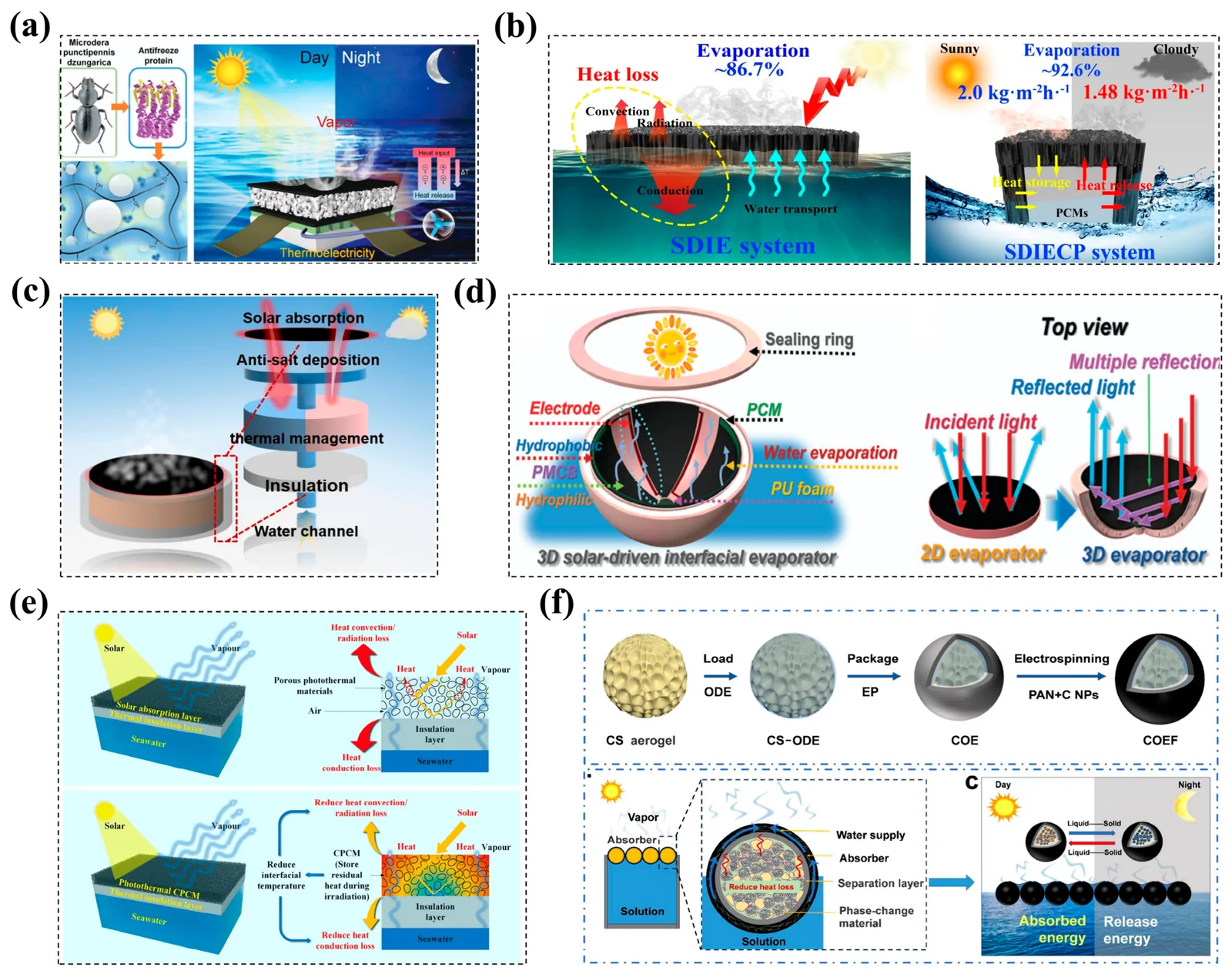
Macroscopic structural designs and thermal-mass flow decoupling mechanisms for phase-change-coupled solar evaporators. (**a**) Configuration and day–night all-weather working mechanism of a biomimetic sandwich-structured solar evaporator inspired by beetles with antifreeze proteins. Reprinted with permission from ref. [53]. Copyright 2023, Wiley-VCH; (**b**) energy efficiency and heat flux modulation comparison between a traditional SDIE system and a SDIECP system under sunny and cloudy conditions. Reprinted with permission from ref. [54]. Copyright 2023, Elsevier; (**c**) schematic structural decomposition of a multi-layered hierarchical configuration based on NiCo_2_O_4_ nanoflowers integrating solar absorption, thermal management, insulation, and water transport functions. Reprinted with permission from ref. [52]. Copyright 2024, Elsevier; (**d**) exploded configuration and top-view light-trapping mechanism of a 3D-printed circular concave solar-driven interfacial evaporator integrated with internal PCMs. Reprinted with permission from ref. [55]. Copyright 2025, Wiley-VCH; (**e**) comparative heat-loss analysis between conventional bilayer systems and integrated photothermal CPCM systems. Reprinted with permission from ref. [56]. Copyright 2026, Elsevier; (**f**) stepwise fabrication protocol of COEF and the corresponding day–night energy absorption/release pathways. Reprinted with permission from ref. [57]. Copyright 2024, Elsevier.

The core design philosophy of sandwich-like and multi-layered hierarchical architectures centers on “functional zoning and spatial decoupling”. In this configuration, the highly absorbent photothermal layer is positioned at the absolute top to directly interface with solar radiation, while the PCM thermal storage layer is enclosed within a porous matrix as an intermediate layer or an independent module, and high-flux water transport channels are interleaved or arranged around the periphery of the thermal storage layer. This physical isolation skillfully constructs a thermal diode-like energy management network: under daytime illumination, excess heat from the top layer is conducted downward to be captured and stored as latent heat by the intermediate PCM layer, restricting invalid dissipation into the bulk water; conversely, under diminished or zero solar irradiance, the latent heat released by the intermediate layer backwardly feeds the top evaporation interface, significantly minimizing thermal losses to the ambient environment. Recently, breakthroughs have been achieved in utilizing such sandwich configurations for waste heat recovery and localized thermal management. For instance, inspired by beetles surviving in extreme climates via antifreeze proteins on their elytra, Niu et al. [53] designed a biomimetic sandwich-structured solar evaporator for all-day operation. This device configuration consists of an intermediate phase change microcapsule/hydrogel composite thermal storage layer sandwiched between top and bottom layers of modified manganese dioxide (MnO_2_) cotton cloth used for synchronous photothermal conversion and capillary wicking. Within this multilayered architecture, the intermediate layer demonstrates superb, localized temperature regulation under 1 kW·m^−2^ illumination, while its latent heat release in the dark sustains an interfacial evaporation rate 3.6 times higher than that of pure water (Figure 4a). A similar spatial heat flux modulation mechanism has been validated in rigid aerogel systems. Gao et al. [54] developed a sandwich-structured MXene/wood aerogel evaporator, embedding the PCM layer as an independent waste heat recovery module within hierarchically porous wood aerogel (Figure 4b). The intrinsic multi-stage longitudinal microchannels of the wood aerogel guarantee high-efficiency water transport and ultra-low thermal conduction, while the interleaved PCM maximally impedes downward heat loss from the MXene photothermal layer, achieving an exceptional energy efficiency of 92.6% under simulated solar irradiation. Additionally, Cheng et al. [52] assembled a novel sandwich evaporator by compounding nanoflower-like transition metal oxides (NiCo_2_O_4_) with a wideband absorption spectrum onto filter paper as the top solar absorber, utilizing a sodium alginate (SA) hydrogel as the intermediate rapid water supply and thermal insulation lining, and integrating a bottom bulk PCM. This setup delivered significant localized heat localization during practical outdoor testing, demonstrating the versatility and superiority of hierarchically decoupled spatial designs for engineering applications (Figure 4c).

Although sandwich hierarchical structures effectively obstruct downward thermal dissipation, the high density and compact texture of the PCM layer in planar layer configurations often compress water transport pathways. This creates a severe mass transfer resistance conflict characterized by abundant thermal energy but insufficient water replenishment, or sluggish vapor escape. To simultaneously attain a high thermal storage capacity and high-flux fluid transport, the implementation of 3D porous frameworks and spatially directional channel structures has become imperative. A 3D skeleton architecture not only vastly expands the gas–liquid evaporation area but also enables low-resistance capillary wicking of water molecules alongside unhindered escape of gaseous vapor via spatial topological tuning, synergistically regulating the energy output of the entire phase change system from a macroscopic mass transfer perspective. Leveraging 3D printing techniques and porous configuration designs allows for the precise reconfiguration and matching of optofluidic and thermal-mass transfer networks. For instance, Li et al. [55] developed a hierarchically structured 3D solar evaporator featuring a 3D-printed circular concave supporter wrapped in a carbon-black-nanoparticle-modified hydrophilic/hydrophobic alternating polyester fabric, with a paraffin-type PCM ingeniously integrated inside (Figure 4d). The circular concave 3D geometry exhibits a unique light-trapping effect capable of harvesting ambient scattered light from multiple angles, while the long-range continuous porous network distributed inside the 3D supporter serves as a high-efficiency water transport highway, flawlessly circumventing the non-hydrophilic bottleneck of the paraffin PCM. This 3D evaporator achieved an anomalously high evaporation rate of 4.0 kg m^−2^·h under natural solar illumination, validating the immense potential of integrating 3D geometries with porous networks to fortify thermal-mass delivery. Furthermore, biomass-derived 3D skeletons and self-floating porous aerogels can optimize heat and mass transfer through the directional alignment of microchannels. Shang et al. [56] synthesized a composite phase change material system (CPCM) using a biomass-derived 3D carbon foam (CF) framework infused with MXene and vacuum-impregnated with liquid PCM. Utilizing the natural, continuous 3D open-cell network of the carbon foam, the system provides both superior thermal regulation stability and drastically accelerated vapor diffusion enabled by its high porosity (Figure 4e). Additionally, Teng et al. [57] fabricated a chitosan (CS)-based 3D self-floating phase change aerogel evaporator, employing directional freeze-drying to impart vertically aligned microchannels that encapsulated an octadecane core, with an electrospun photothermal fiber layer sprayed in situ on the outermost surface (Figure 4f). These vertically oriented pore structures act as a spatial water pump, rapidly pumping water molecules upward against gravity to the surface via strong capillary forces, while the latent heat stored inside the porous matrix sustainably preheats the water stream flowing through the channels. This holistic 3D spatial thermal-mass management thoroughly eliminates localized overheating and dry-out phenomena common in planar configurations, thereby guaranteeing an uninterrupted and continuous evaporation process across both temporal and spatial domains (Table 2).

## 4. Energy Relay and Mass Transfer Control Under Extreme Conditions

The integration of PCMs establishes a temporal “thermodynamic buffer” to overcome the solar intermittency inherent in conventional interfacial evaporators. By storing excess photothermal energy under intense sunlight and releasing latent heat during off-peak or nocturnal hours, PCMs create a continuous energy relay. Beyond sustaining uninterrupted freshwater production, this delayed thermal release maintains a persistent interfacial temperature gradient that fundamentally reconfigures boundary-layer hydrodynamics, effectively overcoming mass-transfer bottlenecks such as salt crystallization and fouling during hypersaline wastewater treatment.

The temporal energy compensation effect of the PCM manifests directly as an anomalous, sustained evaporation behavior under nighttime or zero-light conditions, which substantially amplifies the total daily water yield. Evaporation of conventional systems in the dark relies purely on natural ambient convection, yielding an extremely low rate. Upon integrating a PCM, the energy dissipation process of the system in the dark is markedly retarded. Theoretical and experimental investigations reveal that the phase-change hysteresis effect inside the PCM is the key to achieving steady thermal compensation. To rigorously quantify the energetic performance of thermal management, the daytime thermal storage efficiency ($\eta_{s}$) and the nocturnal heat release efficiency ($\eta_{r}$) can be defined as:

$$\eta_{s}=\frac{m_{PCM}\cdot ∆H_{m}+\int_{T_{0}}^{T_{m}}m_{PCM}C_{p}dT}{I\cdot A\cdot ∆t}$$

$$\eta_{r}=\frac{m_{water,\;\;dark}\cdot h_{lv}}{∆H_{solidification}}$$

where $m_{PCM}$ is the PCM mass, $∆H_{m}$ is the latent heat of fusion, I is solar intensity, A is the evaporator area, $m_{water,\;dark}$ is the cumulative dark freshwater mass, and $h_{lv}$ is the latent heat of water vaporization. Quantitative evaluations indicate that optimized PCM-coupled evaporators store 15–28% of daytime incident solar energy, which subsequently drives nocturnal dark evaporation with heat release efficiencies $\eta_{r}$ exceeding 70–88%.

**Figure 5:**
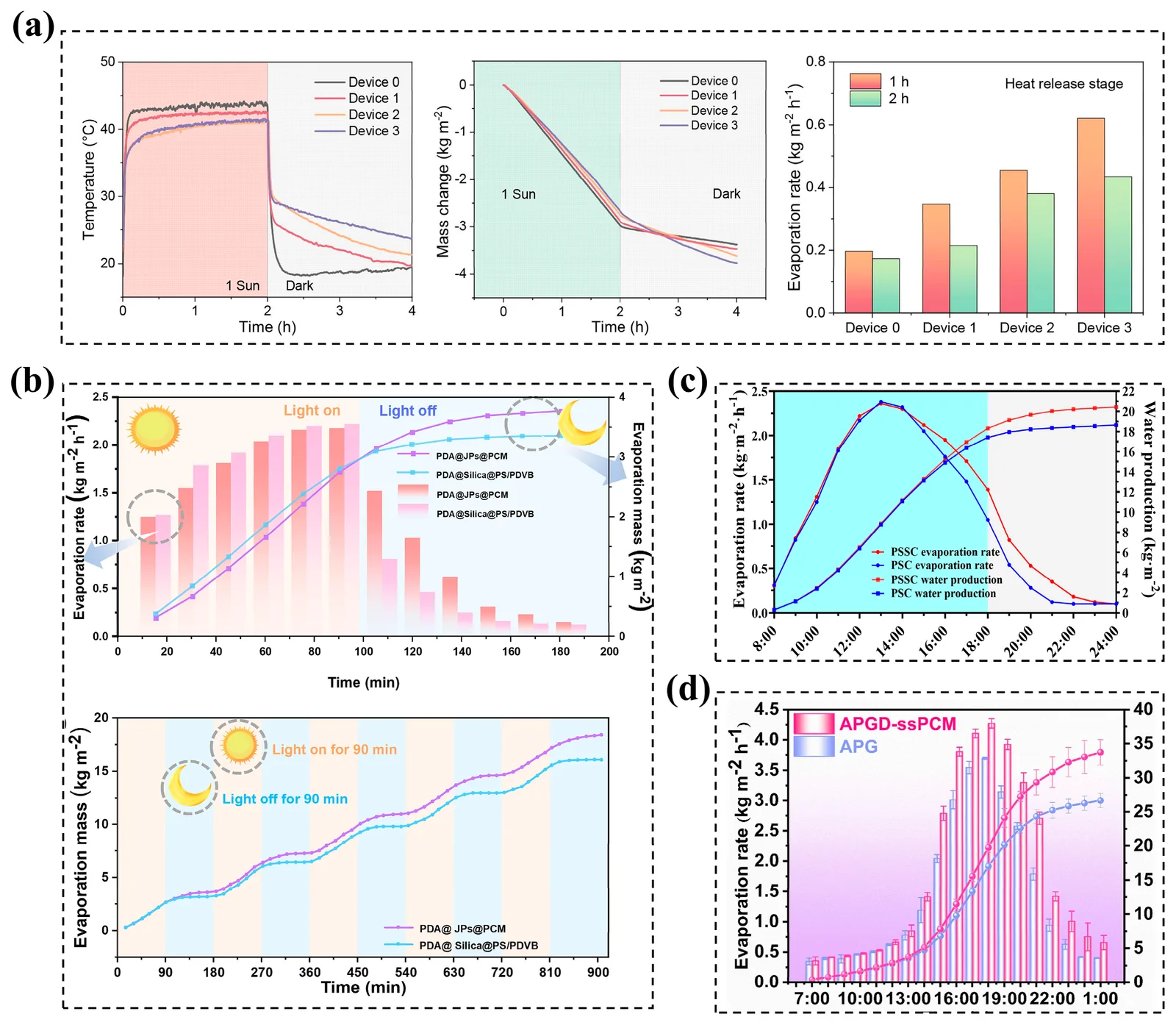
Temporal energy relay performance and all-weather continuous evaporation kinetics of phase-change-coupled interfacial evaporators under light–dark transitions. (**a**) Surface temperature profiles, mass changes during the light–dark cycle, and comparison of the corresponding dark evaporation rates during the heat release stage for different device configurations. Reprinted with permission from ref. [58]. Copyright 2024, Elsevier; (**b**) dynamic real-time evaporation rates and cumulative mass changes under a single light–dark cycle and consecutive multi-cycle alternating illumination states comparing PDA@JPs@PCM and the control group. Reprinted with permission from ref. [49]. Copyright 2026, Elsevier; (**c**) diurnal outdoor variations of water production and real-time evaporation rates for the PSSC hydrogel system and the PSC control group. Reprinted with permission from ref. [59]. Copyright 2025, Elsevier; (**d**) diurnal time-dependent evaporation rate profiles and long-term continuous water collection behavior comparing APGD-ssPCM and APG. Reprinted with permission from ref. [51]. Copyright 2025, Elsevier.

Lan et al. [58] discovered that in a paraffin-integrated solar evaporation system, although the daytime diversion of partial heat into the paraffin layer slightly compromised the instantaneous daytime evaporation rate, the delayed phase-change heat release at various internal locations triggered a dramatic 171.5% surge in nocturnal dark evaporation, culminating in an 11.5% net increase in the total cumulative water mass after one complete light–dark cycle. In some classical energy balance analyses, the integrated PCM evaporator across the entire cycle of light-to-thermal conversion and waste heat storage accounts for a mere 5.2% of the incident solar energy. It delivers an evaporation rate as high as 0.70 kg·m^−2^·h^−1^ and an energy efficiency of 46.5% under dark conditions, an efficiency that is 2.5 times higher compared to conventional evaporators (Figure 5a). Ma et al. [49] demonstrated that by introducing Janus phase change microcapsules (PDA@JPs@PCM) or hollow microsphere sandwich structures, the total freshwater yield of the system under extremely unstable solar intensities, such as cloudy or overcast conditions, could be reliably enhanced by 1.24 kg·m^−2^. Benefiting from the latent heat release of the octadecane core, the system sustained continuous evaporation in dark environments, accumulating a total water output of 18.41 kg·m^−2^ over five consecutive light–dark cycles (surpassing the 16.08 kg·m^−2^ of the control group) and highlighting a more outstanding reusability and comprehensive photothermal storage/release performance (Figure 5b). Under real-world complex outdoor meteorological conditions, this “peak-shaving and valley-filling” capability exhibits profound engineering practicality. For example, Chen et al. [59] distributively packaged sodium sulfate decahydrate within a sodium alginate/polyacrylamide dual-network hydrogel using commercial ink as the photothermal absorber, successfully constructing an all-weather interfacial evaporation system (PSSC) that harmonizes highly efficient photothermal conversion with high heat-storage capacities. In field-scale outdoor desalination trials, the PSSC system achieved an actual cumulative daily freshwater yield as high as 20.43 kg·m^−2^, a significant increase of 1.77 kg·m^−2^ compared to the hydrogel control group lacking phase change materials (Figure 5c). Concurrently, composite systems utilizing solid–solid phase change energy storage gels (such as APGD-ssPCM) reached an ultra-high daily water collection of 33.72 kg·m^−2^ in outdoor tests, noticeably outperforming the 26.67 kg·m^−2^ of the non-PCM counterpart, while maintaining an evaporation rate of 0.97 kg·m^−2^·h^−1^ even when held in a dark environment for 6 h [51] (Figure 5d). Correspondingly, biomimetic sandwich-structured PCM/hydrogel systems or graphene-paraffin-carbon nanotube radiant heating designs can achieve dark evaporation rates over 3.6 times higher than that of pure water, or extend the effective evaporation duration by approximately 38% [53]. These data collectively demonstrate that PCM-coupled interfacial evaporation systems possess immense engineering potential for all-weather, full-cycle continuous water harvesting via the time-delayed release of latent heat.

In continuous open-sea desalination and hypersaline industrial wastewater zero liquid discharge (ZLD) treatments, severe localized concentration of brine leading to surface salt crystallization and clogging is a devastating bottleneck limiting the long-term operational durability of evaporators [60]. Traditional anti-salt designs primarily rely on passive diffusion during illuminated daytime hours [61,62,63]. However, after sunset, the sudden drop in surface temperature causes the thermal localization effect to vanish, which abruptly weakens the diffusion kinetics of salt ions at the surface layer; the highly concentrated brine accumulated during the day then undergoes rapid saturation crystallization under low temperatures, quickly choking the porous channels. The introduction of PCM-coupled systems thoroughly rewrites this process from the standpoint of underlying heat and mass transfer kinetics. When solar irradiance shifts to the dark state, the latent heat released from the embedded PCM drives a backward thermal conduction from the interior to the exterior, thereby sustaining a subtle yet long-lasting micro-temperature gradient at the gas–liquid evaporation interface. This microscopic thermal gradient triggers two critical mass transfer phenomena within the fluid boundary layer: first, the sustained existence of Marangoni convection (surface tension-driven flow), where the localized surface tension gradient induced by the temperature difference generates a downward micro-shear force that actively accelerates the backflow of concentrated surface brine into the underlying lower-concentration bulk water [64]. Second, it thermodynamically activates thermophoresis and ion diffusion coefficients; maintaining a higher interfacial temperature significantly elevates the diffusion coefficient of salt ions, overcoming the crystallization tendency stemming from concentration polarization under cold states [65]. In recently reported tests under extreme operating conditions, this continuous spatiotemporal mass-transfer management has demonstrated astonishing anti-crystallization capabilities. Clarifying the distinct operating regimes and relative thermodynamic contributions of Marangoni convection and thermophoretic diffusion is crucial for understanding boundary-layer hydrodynamics [66,67,68]. Marangoni convection is a macro/meso-scale fluid dynamic phenomenon driven by surface tension gradients ($\nabla \gamma =\frac{\partial \gamma }{\partial T}\nabla T+\frac{\partial \gamma }{\partial C}\nabla C$) across millimeter-to-centimeter domains. During nocturnal latent heat release, the persistent temperature difference between the warm evaporator surface and cooler surrounding water induces micro-vortices, generating downward hydrodynamic shear forces that actively pump concentrated surface brine back into bulk water [69]. Conversely, thermophoretic diffusion operates as a micro/nano-scale mass transfer mechanism governed by the thermal diffusion flux ($J_{thermo}=-\rho D_{T}\nabla T$) across sub-millimeter boundary layers and micropores. Under persistent thermal gradients sustained by PCMs, thermophoresis drives hydrated salt ions away from the warm evaporation interface at the molecular level, suppressing concentration polarization prior to salt nucleation. 

**Figure 6:**
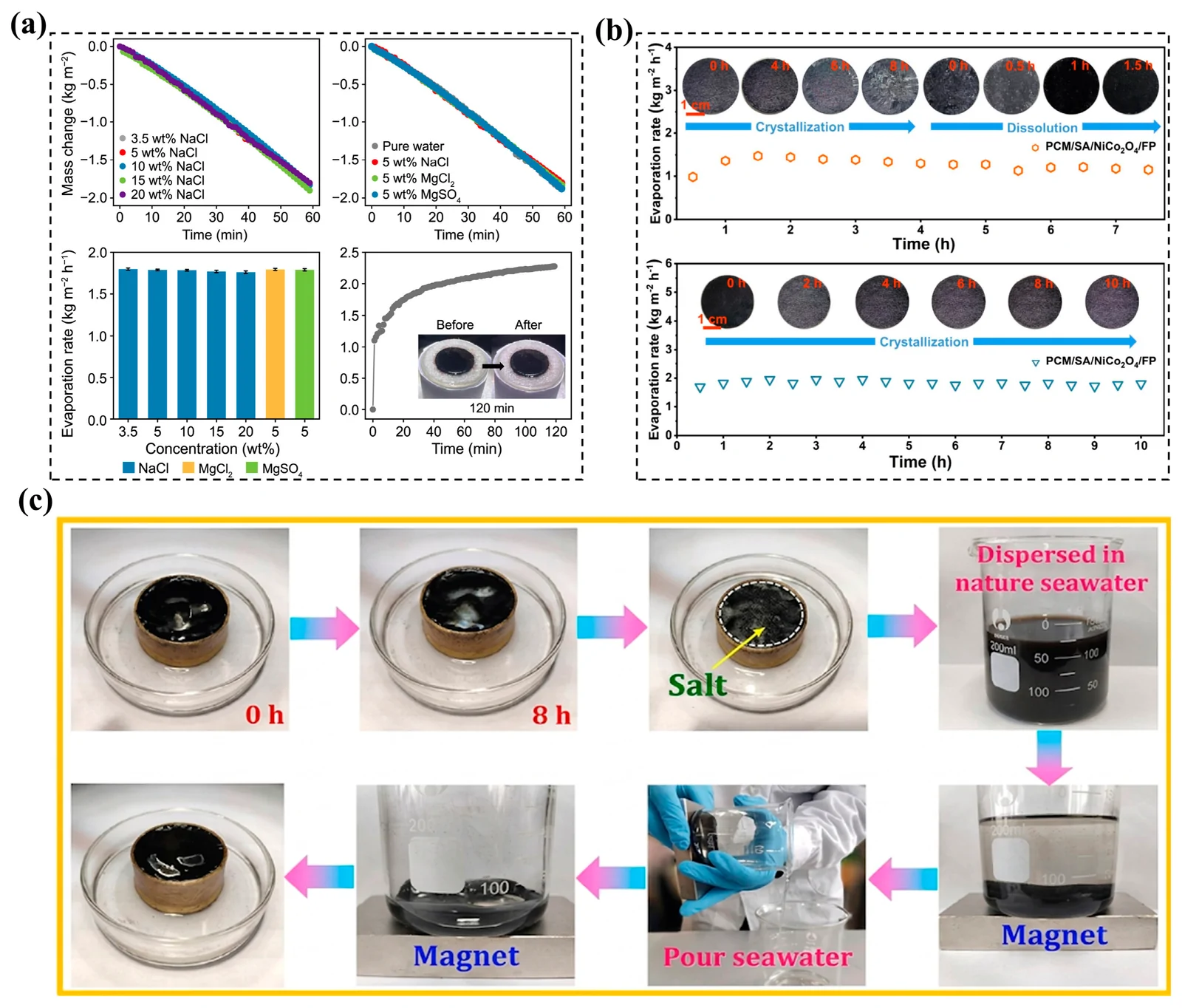
Anti-salt crystallization performance, self-cleaning mechanisms, and magnetic-assisted recycling of phase-change-coupled evaporators under hypersaline environments. (**a**) Mass changes and steady evaporation rates under different concentrations and salt types, alongside surface photographs showing the evaporator status before and after operation. Reprinted with permission from ref. [71]. Copyright 2026, Elsevier; (**b**) evaporation rates and corresponding time-lapse surface photographs of the PCM/SA/NiCo_2_O_4_/FP sandwich system. Reprinted with permission from ref. [52]. Copyright 2024, Elsevier; (**c**) photographic demonstration of the magnetic-responsive separation, dispersion, washing, and damage-free recovery process of the magnetic phase-change evaporator matrix guided by an external magnetic field. Reprinted with permission from ref. [48]. Copyright 2022, American Chemical Society.

Furthermore, long-term operational stability under prolonged hypersaline conditions (>100 h continuous operation) remains a vital benchmark. Under continuous high-flux evaporation, evaporators face severe contact angle hysteresis and scaling [70]. In PCM-coupled systems, periodic nocturnal dark cycles allow sustained thermal release to maintain ion back-diffusion while dynamic re-dissolution dissolves minor salt crystals formed during daytime peaks, fully restoring capillary pathways for long-term operational durability. Zhou et al. [71] developed a multi-functional composite phase change foam by confining dodecylamine within a polypyrrole-coated chitosan–phenolic network, which achieved a stable, continuous evaporation rate of 1.862 kg·m^−2^·h^−1^ under 1 sun and maintained 0.684 kg·m^−2^·h^−1^ in the dark even when processing an extremely high concentration of 20 wt.% NaCl simulated industrial brine. Remarkably, the evaporator surface remained completely pristine without any visible salt crystal accumulation during 10 h of outdoor solar desalination (Figure 6a). Correspondingly, evaporators based on hierarchical MXene/wood aerogel composite PCMs or sandwich configurations embedded with NiCo_2_O_4_ nanoflowers can sustain continuous, salt-free desalination for 5 to over 9 days when confronted with hypersaline environments [52] (Figure 6b). Furthermore, addressing the inevitable crystal precipitation at the later stages of specific salt-lake or hypersaline wastewater evaporation, recent material engineering has introduced physical isomorphic designs to realize rapid brine removal and resource recovery. For example, by integrating magnetic nanoparticles within the microcapsule shells, the evaporation material itself is endowed with magnetic response capabilities [48]. Following continuous high-intensity operation in a 10 wt.% hypersaline NaCl solution, even if a minor amount of salt crystals precipitates on the surface, the entire phase change evaporation matrix can be effortlessly separated via simple washing coupled with an external magnetic field for rapid, damage-free recovery (Figure 6c). This fundamentally bypasses the structural scrap dilemma of conventional immobilized evaporators post-crystallization, perfectly uniting thermodynamic latent-heat-controlled crystallization with kinetic magnetic separation to establish a solid operational foundation for implementing industrial-scale all-weather zero liquid discharge treatments.

## 5. Cross-Disciplinary Integration: Multifunctional System Integration

Following the circumvention of shape-stability bottlenecks and the realization of efficient spatiotemporal thermal-mass management via micro-to-macro structural engineering, the application boundaries of phase-change-coupled interfacial evaporation systems have transcended the singular paradigm of freshwater harvesting [72,73,74]. During open evaporative operations, the photothermal interface not only accumulates a massive amount of low-grade thermal energy but also drives a pronounced ion concentration gradient and a localized solute enrichment zone within the surface fluid layer as water vapor dissipates [75,76]. This unique microenvironment affords an exceptional physicochemical platform for complementary multi-energy harvesting, in-situ recovery of target resources, and accelerated chemical catalysis [77,78]. The core contribution of integrating PCMs into these multifunctional configurations lies in their role as an underlying thermodynamic regulator. The stable and long-lasting latent heat released by the PCM effectively dampens environmental temperature fluctuations and hydrodynamic oscillations triggered by meteorological variations and diurnal transitions, thereby ensuring the stable output of water–electricity co-generation devices and the uninterrupted progress of chemical reactions.

**Figure 7:**
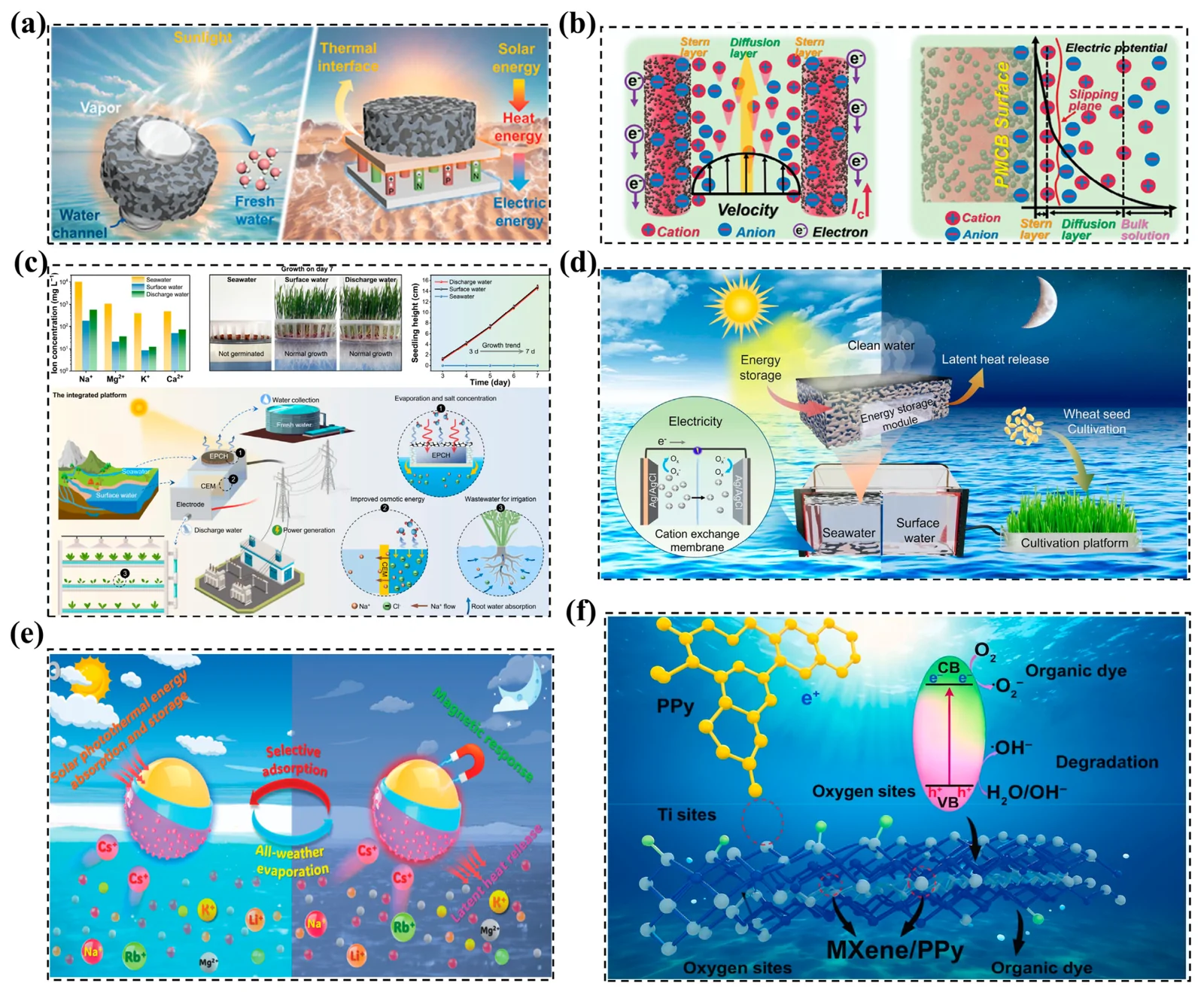
Cross-disciplinary integrations and multifunctional applications of phase-change-coupled solar interfacial evaporation platforms. (**a**) Schematic configuration of simultaneous SDIE and TEG for water–electricity co-generation. Reprinted with permission from ref. [79]. Copyright 2024, Wiley-VCH; (**b**) electrokinetic/salinity gradient energy harvesting mechanisms showing the Stern layer, diffusion layer, fluid velocity profile, and electric potential distribution at the evaporation interface. Reprinted with permission from ref. [55]. Copyright 2024, Wiley-VCH; (**c**) layout and experimental crop-growth validation of the integrated WEF nexus platform powered by encased phase-change hydrogels for continuous desalination, power generation, and irrigation. Reprinted from ref. [80]; (**d**) all-weather operational pathway of the intelligent micro-system combining phase-change energy storage modules, ion exchange membranes, and wheat seed cultivation. Reprinted from ref. [81]; (**e**) selective extraction mechanism and magnetic-assisted recovery of Cs^+^ from complex brine via crown-ether-decorated phase-change microcapsules under day–night alternating cycles. Reprinted with permission from ref. [82]. Copyright 2024, Wiley-VCH; (**f**) energy band structure and photothermal–photocatalytic synergistic mechanism for the in-situ degradation of organic dyes at the MXene/PPy heterojunction interface. Reprinted from ref. [83].

The hybridization of interfacial evaporation with secondary energy co-generation (e.g., water–electricity co-generation) represents an effective strategy to alleviate the concomitant global crises of energy and water scarcity. Currently, the prevailing co-generation mechanisms are categorized into thermoelectric generation (TEG) driven by temperature differentials and reverse electrodialysis (RED) powered by salinity gradients. However, owing to the intrinsic intermittency of natural solar irradiation, conventional co-generation devices typically exhibit highly volatile, pulse-like output voltages and power densities, rendering them unsuitable for directly powering electronic components. The introduction of PCMs flawlessly circumvents this output volatility through the microscopic regulation of the thermodynamic boundary conditions of the system. In the realm of TEG co-generation, the PCM primarily serves to sustain the critical temperature gradient across the thermoelectric modules under dark conditions. Recent advancements demonstrate that Tang et al. [79] engineered a composite system utilizing amino-attapulgite/graphene hybrid aerogels to encapsulate lauric acid (LA/GNAb). By establishing continuous internal heat conduction pathways, this configuration not only achieved a high evaporation rate of 2.13 kg·m^−2^·h^−1^ but also delivered a stable thermoelectric power density of 1.57 W·m^−2^ under 1-sun illumination, successfully extending the electricity output duration into the dark state (Figure 7a). Similarly, a biomimetic sandwich-structured PCM/hydrogel system coupled with a thermoelectric module demonstrated a stable electrical output of 0.42 W·m^−2^ during the day and sustained electricity generation for up to 30 min after sunset, representing a multi-fold enhancement in dark operational duration compared to conventional non-thermal storage counterparts [53]. Furthermore, Li et al. [55] deeply integrated a paraffin-type PCM with a hierarchical 3D circular concave supporter possessing a light-trapping architecture. By leveraging the abundant latent heat stored within the PCM, the hybrid device dramatically reduced the capacitor charging time under intermittent solar irradiation by 57.9% and achieved a maximum output voltage of 3.51 V, validating the profound superiority of PCMs as thermal source regulators in dampening thermoelectric output pulses (Figure 7b).

In the direction of salinity gradient energy harvesting via RED and its integration with the water–energy–food (WEF) nexus, PCM-coupled systems safeguard the long-term sustainability of the high-concentration salinity gradient by stably maintaining the interfacial evaporative concentration rate. For instance, Zhang et al. [80] engineered an encased phase-change hydrogel (EPCH) system by embedding phase-change microcapsules within a polyurethane sponge matrix, enhancing the solar utilization efficiency by 27%. Concurrently, the EPCH not only achieved highly efficient desalination but also boosted the power density of the coupled RED system by an astonishing 146% by stably maintaining a high localized salinity at the gas–liquid interface (Figure 7c). This platform uniquely integrates self-adaptive thermal regulation, anti-fouling resistance against salt and oil, evaporation-driven salinity control, and synchronous freshwater–electricity co-generation within a unified matrix, successfully implementing water–power co-generation while utilizing the discharged water for crop cultivation. Building upon this concept, Wang et al. [81] further designed an integrated, intelligent micro-system that unifies freshwater production, salinity gradient power generation, and crop irrigation. Utilizing a phase-change microcapsule/hydrogel system optimized for thermal management, the platform sustained a nocturnal dark evaporation rate of approximately 0.54 kg·m^−2^·h^−1^ and continuous RED power generation of ~0.3 W·m^−2^, while directly channeling the secondary pollution-free, nutrient-rich concentrated wastewater to a downstream automated soilless cultivation platform for real-time, on-demand wheat irrigation (Figure 7d). This breakthrough signifies that PCM-coupled configurations have officially transcended the preliminary phase of pure materials research and are evolving into green engineering platforms featuring full-time, multi-ecological element coupling.

Concomitant with the sophisticated refinement of multifunctional evaporators, exploiting the physical concentration effect during the interfacial evaporation process to achieve the green extraction of high-value elements or the in-situ degradation of recalcitrant organic pollutants in wastewater has become another cutting-edge frontier in material design. The thermal storage properties of PCMs offer unique kinetic advantages for both catalysis and adsorption in these applications: by maintaining the interfacial reaction system in a highly thermally activated state at night, they prevent catastrophic drop-offs in catalytic reaction rates or adsorption kinetics triggered by precipitous nighttime temperature declines. In the context of the highly efficient recovery of specific mineral resources, functionalizing the phase-change shell with targeted chemical groups represents an innovative strategy. For example, addressing the high-value yet exceedingly difficult-to-separate alkali metal ions within salt-lake brine, Zhang et al. [82] fabricated an integrated evaporator based on crown-ether-decorated phase-change microcapsules (Crown-MicPCM). This material encapsulates an n-docosane core for latent heat storage within a shell modified through meticulous multi-layer functionalization, which isomorphically integrates light-absorbing polydopamine (PDA) with crown ether molecules possessing specific spatial matching effects. Throughout the evaporative desalination of salt-lake water, the rapid escape of water vapor induces a swift enrichment of Cs^+^ ions at the surface layer. Concurrently, the shell-decorated crown ethers immediately execute in-situ lattice capture of the concentrated target ions, achieving an outstanding selective Cs^+^ adsorption capacity of 32.6 mg·g^−1^. Throughout this process, the long-lasting thermal environment provided by the PCM significantly elongates the thermal motion trajectories of the molecules, leading to a counter-trend 12% enhancement in Cs^+^ extraction efficiency compared to control groups lacking a phase-change core (Figure 7e). In the domain of in-situ treatment of highly recalcitrant wastewater and environmental remediation, PCM-coupled configurations similarly manifest prominent photothermal-photocatalytic synergistic effects. Chen et al. [83] developed a directionally structured alginate aerogel evaporator (OSLA-MXene/PPy), where the lower skeleton was doped with silica hollow spheres encapsulating an octadecylamine phase-change core, while the top layer was crosslinked with Ti_3_C_2_Tx MXene nanosheets and PPy layers exhibiting superior degradation performance. When treating textile printing and dyeing wastewater, the top layer executes broadband spectral absorption and photo-excites highly active electron-hole pairs to generate a profusion of active free radicals in situ, while the latent heat stored within the underlying phase-change core backwardly maintains the activation energy requirements of the catalytic interface during dark or low-light periods. Experimental evaluations revealed that while achieving a highly efficient evaporation rate of 1.62 kg·m^−2^·h^−1^, this synergistic system reached astonishing in-situ catalytic removal efficiencies of 99.54% and 84.43% for recalcitrant Rhodamine B and Methylene Blue, respectively (Figure 7f). Furthermore, within solid–solid phase change frameworks based on BC/PVA hydrogels, PEG/PAA composite networks, and hollow polymer microsphere hybrid phase-change systems, investigators have similarly substantiated that the dynamically balanced heat flux sustained by the PCM endows the reaction interface with exceptional chemical stability and anti-fouling self-cleaning traits when exposed to harsh industrial dyeing effluents and corrosive chemical acid-base solutions [50]. This integrated architecture perfectly achieves the spatiotemporal isomorphism of physical thermal-storage evaporation and chemical in-situ degradation, offering a low-carbon, highly efficient paradigmatic blueprint for all-weather green industrial wastewater remediation. Despite these multi-energy synergies, integrating multifunctional capabilities inevitably introduces thermodynamic energy-budget trade-offs. The total absorbed broadband solar heat ($Q_{abs}$) is governed by an overarching energy balance ($Q_{abs}=Q_{evap}+Q_{TEG/RED}+Q_{catalysis}+Q_{loss}$). Maximizing thermoelectric generation requires establishing a large temperature gradient across the thermoelectric module, which intentionally channels a portion of heat away from the gas–liquid interface, thereby reducing instantaneous daytime freshwater evaporation rates by 10–22%. Similarly, leveraging interfacial evaporation to induce high salinity gradients for reverse electrodialysis or targeted ion extraction requires elevating surface solute concentration; however, excessive salt enrichment increases the thermodynamic chemical potential barrier for water vaporization and heightens salt crystallization risks [84]. Thus, system architects must rationally balance energy splitting according to targeted application priorities.

## 6. Conclusions

Integrating PCMs into SDIE systems marks a system-level paradigm shift rather than a mere incremental optimization, offering a robust strategy to overcome solar intermittency and weather volatility. Microscopically, advanced encapsulation frameworks incorporating 3D matrix confinement, core–shell architectures, and covalent solid–solid transitions fundamentally reconfigure interfacial heat transfer networks. These strategies completely eliminate molten media leakage and secondary water contamination while effectively suppressing supercooling and boosting thermal conductivity. Macroscopically, hierarchical sandwich configurations and directional porous channels achieve distinct spatial decoupling of water supply, photothermal conversion, and thermal storage. This enables a cross-timeline “energy relay” via the highly controlled release of stored latent heat, ensuring industrial-grade, all-weather continuous evaporation. Furthermore, the latent heat-sustained temperature gradient continuously drives interfacial Marangoni convection and thermophoretic diffusion, hydrodynamically preventing salt accumulation and achieving long-term ZLD in hypersaline environments. Serving as a thermodynamic regulator against weather fluctuations, this spatiotemporal thermal-mass management platform successfully propels SDIE beyond freshwater harvesting into co-generation, targeted resource recovery, and environmental remediation.

To accelerate material discovery and conquer the multiscale thermal-mass transport complexities in phase-change-coupled SDIE systems, integrating physics-informed machine learning, quantitative sensitivity analysis, digital twins, and standardized evaluation protocols represents a transformative frontier [85]. Seamlessly fusing data-driven algorithms (e.g., GNNs, Bayesian optimization, neural operators) with physical conservation laws via Physics-Informed Neural Networks (PINNs) provides a thermodynamically consistent, mesh-free solver to rapidly calculate complex boundary-layer thermal-mass flow fields [86]. Concurrently, incorporating quantitative sensitivity analysis and Uncertainty Quantification (UQ) systematically evaluates the non-linear coupling and relative significance of key design parameters—such as PCM latent heat, thermal conductivity, encapsulation shell thickness, and environmental volatility—on long-term operational durability [87]. Coupling these AI paradigms with real-time Digital Twins will enable dynamic thermal-mass monitoring, predictive self-cleaning, and automated fault diagnosis in field operations. Ultimately, establishing standardized performance evaluation metrics—including normalized solar irradiance protocols, standardized brine concentrations, and unified definitions for thermal storage/release efficiencies ($\eta_{s}$ and $\eta_{r}$)—will close the loop between multiscale simulation, intelligent optimization, and industrial deployment, driving phase-change-coupled SDIE technology toward a standardized, scalable, and intelligent reality.

## Figures and Tables

**Table 1:** Systematic comparison of microscopic encapsulation strategies for SLPCMs in SDIE systems.

Encapsulation Strategy	Working Mechanism and Interfacial Physics	Thermal and Mass Transport Characteristics	Advantages and Limitations	Representative Examples and Key Metrics
3D Matrix Confinement	Physical trapping via capillary forces and hydrogen bonding within 3D porous networks (aerogels, hydrogels, biomass frameworks).	Reduces bulk liquid fluidity; thermal conductivity enhanced by conductive fillers; moderate mass resistance depending on pore size.	Pros: Simple fabrication, low cost, scalable, versatile substrate choice. Cons: Potential contact thermal resistance; slight leakage under high-stress cycling.	C-EVOH-PEG: 1.77% leakage loss, 189.7 J·g^−1^ [41] EG-Co_3_O_4_/PEG: 6.43 W·m^−1^·K^−1^, efficiency increased to 80.3% [43] AM/PEG/BC/CMC@PDA [44]
Core–Shell Architectures	Physical isolation of PCM core within an inorganic (SiO_2_, TiO_2_) or hybrid polymeric shell via microencapsulation.	Eliminates direct solvent contact; shell can be functionalized with photothermal (MXene, PDA) or magnetic materials; localized thermal response.	Pros: Superior anti-leakage stability, magnetic recyclability, tunable shell functions. Cons: Shell adds non-phase-change dead weight; complex multi-step synthesis.	SiO_2_/Fe_3_O_4_@PPy/MXene [47] MXene/PDA@TiO_2_/Fe_3_O_4_@n-docosane: >95% light absorption, +1.17 kg·m^−2^ dark yield [48] PDA@JPs@PCM Janus microcapsules [49]
Covalent Solid–Solid Transitions	Chemical grafting of phase-change segments as side chains/blocks onto rigid backbones; state transition between crystalline and amorphous.	Zero liquid phase formation; intrinsic thermal resistance minimized; co-functionalized hydrophilic channels facilitate ultra-fast water pumping.	Pros: Absolute zero leakage under extreme acid/alkali/organic conditions, exceptional structural durability. Cons: Synthetic complexity; slightly lower latent heat density.	BC/PVA/GO-g-LA SSPCM: zero leakage after 15 extreme light-dark cycles [50] APGD-ssPCM: 96.2% light absorption, water transport 1.93 g·g^−1^·min^−1^, 33.72 kg·m^−2^ daily yield [51]

**Table 2:** Systematic comparison of macroscopic structural designs for phase-change-coupled evaporators.

Macroscopic Design	Spatial Heat-Mass Decoupling Principle	Thermal Management Efficiency	Advantages and Limitations	Representative Systems and Performance
Sandwich and Multilayer Structures	Top photothermal layer + intermediate PCM thermal storage layer + bottom/peripheral water wicking channels. Functionally separates light absorption from thermal storage.	Acts as a thermal diode; stores excess daytime heat and feeds it backward to the evaporation surface at night, drastically cutting downward conductive loss.	Pros: Excellent thermal localization, high energy efficiency (>90%), straightforward functional zoning. Cons: Dense planar PCM layer may restrict vertical water flow if channels are unoptimized.	Beetle-inspired MnO_2_/PCM sandwich: dark evaporation rate 3.6× pure water [53] MXene/wood aerogel SDIECP: 92.6% efficiency under 1 sun, 1.48 kg·m^−2^·h^−1^ in cloudy conditions [54] NiCo_2_O_4_/SA/PCM sandwich [52]
3D Directional Porous Channels	3D spatial topologies (concave, cylindrical, self-floating aerogels) with anisotropic vertically aligned channels. Integrates capillary pumping directly through PCM matrix.	Omnidirectional light trapping harvested from ambient/scattered light; continuous preheating of flowing water stream; expanded evaporation surface area.	Pros: Solves water-supply bottleneck in dense PCMs, drastically increases mass flux, prevents localized dry out. Cons: Larger physical footprint; requires precision processing (e.g., 3D printing, directional freeze drying).	3D-printed circular concave PCM evaporator: 4.0 kg·m^−2^·h^−1^ under natural sunlight [55] Carbon foam/MXene CPCM: high porosity vapor diffusion [56] CS-based 3D self-floating aerogel (COEF): vertically aligned pumping microchannels [57]

## Data Availability

No new data were created or analyzed in this study. Data sharing is not applicable to this article.
